# Access to a psychiatric emergency setting during the COVID-19 pandemic: focus on youth populations

**DOI:** 10.1192/j.eurpsy.2023.506

**Published:** 2023-07-19

**Authors:** F. Masini, G. Caramanico, G. Menculini, G. Latini, F. De Giorgi, K. Amantini, P. Moretti, A. Tortorella

**Affiliations:** ^1^Department of Psychiatry; ^2^School of Medicine, University of Perugia; ^3^Section of Psychiatry, Clinical Psychology and Rehabilitation, Santa Maria Della Misericordia Hospital; ^4^Psychiatric Inpatient Unit, Department of Mental Health, AUSL Umbria 1, Perugia, Italy

## Abstract

**Introduction:**

The COVID-19 outbreak and the related containment measures led to the emergence of psychological distress in youth populations, possibly due to concern for their families, social isolation, increased time spent on the Internet and social media, and anxiety about the future.

**Objectives:**

The study aims to evaluate differences in the access of children, adolescents, and young adults to a psychiatric emergency setting before and after the onset of the COVID-19 pandemic.

**Methods:**

Data concerning the psychiatric consultations carried out at the Emergency Department of the University Hospital of Perugia was collected. Socio-demographic and clinical information, including diagnostic and treatment features, was entered into an electronic database. We considered two different time spans, one before (01.06.2017-31.12.2018) and one after (01.06.2020-31.12.2021) the COVID-19 pandemic outbreak. The characteristics of consultations carried out before and after the pandemic outbreak were compared by means of bivariate analyses (p<0.05).

**Results:**

2,457 psychiatric consultations were carried out in the index periods. 1,319 (53.7%) were requested before, while 1,138 (46.3%) after the COVID-19 outbreak. As for the latter, these were more frequently requested for female subjects (64.2% vs 54.5%, p=0.0042), while institutionalized people underwent psychiatric consultations less frequently in the post-COVID-19 period (5.6% vs 18.2% p<0.001). A significant difference in the prevalence of anxiety disorders (9.7% post-COVID-19 vs 18.8% pre-COVID-19, p=0.009) and adjustment disorders was found (7.1% vs 1.5%, p=0.009). Substance-related disorders were significantly reduced (8.0% vs 15.8%, p=0.016) after the COVID-19 outbreak. About psychopharmacological treatment, there was an increase in people who had received treatment in the past but were no longer on treatment (52.3% vs 30.8%, p<0.001). The prescription of antipsychotics also increased (29.3% vs 18.5%, p=0.012). At discharge, subjects were more frequently hospitalized in the Psychiatric Inpatient Unit in the post-COVID-19 period (22.2% vs 12.8%, p=0.012).

**Conclusions:**

Our data confirms the vulnerability of youth populations during the pandemic. The consequences of health emergencies on the psychological well-being of this population must not be underestimated and tailored treatment strategies should be implemented.

**Disclosure of Interest:**

None Declared

